# Insights into the Antimicrobial Properties of Hepcidins: Advantages and Drawbacks as Potential Therapeutic Agents

**DOI:** 10.3390/molecules20046319

**Published:** 2015-04-10

**Authors:** Lisa Lombardi, Giuseppantonio Maisetta, Giovanna Batoni, Arianna Tavanti

**Affiliations:** 1 Dipartimento di Biologia, Università di Pisa, Via San Zeno, 37, 56127 Pisa, Italy; E-Mail: lisa.lombardi@for.unipi.it; 2 Dipartimento di Ricerca Traslazionale e delle Nuove Tecnologie in Medicina e Chirurgia, Università di Pisa, Via San Zeno, 37, 56127 Pisa, Italy; E-Mails: gmaisetta@biomed.unipi.it (G.M.); giovanna.batoni@med.unipi.it (G.B.)

**Keywords:** antimicrobial peptides, hepcidins, antibacterial activity, antifungal activity

## Abstract

The increasing frequency of multi-drug resistant microorganisms has driven research into alternative therapeutic strategies. In this respect, natural antimicrobial peptides (AMPs) hold much promise as candidates for the development of novel antibiotics. However, AMPs have some intrinsic drawbacks, such as partial degradation by host proteases or inhibition by host body fluid composition, potential toxicity, and high production costs. This review focuses on the hepcidins, which are peptides produced by the human liver with a known role in iron homeostasis, as well by numerous other organisms (including fish, reptiles, other mammals), and their potential as antibacterial and antifungal agents. Interestingly, the antimicrobial properties of human hepcidins are enhanced at acidic pH, rendering these peptides appealing for the design of new drugs targeting infections that occur in body areas with acidic physiological pH. This review not only considers current research on the direct killing activity of these peptides, but evaluates the potential application of these molecules as coating agents preventing biofilm formation and critically assesses technical obstacles preventing their therapeutic application.

## 1. Introduction

Nowadays, microbial resistance to antibacterial and antifungal drugs has reached critical levels, invalidating the therapeutic efficacy of a large proportion of molecules currently used in the clinic [1,2]. The scenario is particularly alarming in hospitals, where the emergence and rapid spread of microorganisms resistant to diverse classes of antimicrobial drugs (Multidrug-Resistant—MDR—strains) have an enormous impact on morbidity and mortality as well as on healthcare costs [1,2].

The increment in the rate of resistance of clinically relevant microorganisms has paralleled a progressive reduction in the number of antibiotics approved for clinical use over the past 30 years [3]. Therefore, to address this unforeseen threat, extensive research efforts focused on the development of new antimicrobial compounds and new strategies for combating multidrug-resistant microorganisms represent a top priority in medicine [4].

Among future anti-infective drugs under development, antimicrobial peptides (AMPs) have been repeatedly proposed as a valid alternative to antibiotics [5,6,7]. These are a large and diverse group of ubiquitous molecules, referred to as effectors of the innate immune system of organisms belonging to different levels of the evolutionary scale, and involved in the first line of defense against infectious agents [8]. In addition to their generally fast and strong antimicrobial activity, these molecules are often able to modulate immune response, recruit immune cells to the infectious site, and neutralize bacterial endotoxin suggesting that, *in vivo*, besides the direct killing of microorganisms, they can indirectly participate to the eradication of infections [7]. These characteristics, together with the fact that AMPs are less prone to induce the development of resistance compared to conventional antibiotics, render these molecules lead compounds for anti-infective agent development. Several drawbacks, however, still limit AMP clinical use as systemic drugs, while their use as topical agents to treat muco-cutaneous infections seems more feasible.

The present review focuses on the antimicrobial properties of hepcidins, AMPs produced by a large variety of living organisms and endowed with a range of biological activities. In particular, based on our previous studies and findings from other research groups, we provide evidence that human hepcidins display interesting features that suggest the possible use of these molecules, or their optimized derivatives, as future drugs to treat microbial infections of mucosal surfaces with acidic physiological pH.

## 2. Antimicrobial Peptides and Their Potential as Novel Antimicrobial Compounds

AMPs are short (typically 12–100 residues) positively charged (from +2 to +9) molecules, depending on their amino acidic composition [9]. AMPs play a key role in the first line immune defense in a wide range of organisms, including plants, protozoa, arthropods, insects, fish and mammals [10,11]. Depending on their secondary structure, AMPs fall within four main classes; alpha-helical peptides, beta-sheet peptides, extended peptides, and loop peptides [12]. Regardless of the specific structure, several interesting features suggest that AMPs may be a promising class of potential antimicrobial agents. Indeed, AMPs have been proven to exert an antimicrobial action *versus* bacteria, but also *versus* virus, fungi and protozoa; notably, many of them are active against multi-drug resistant bacteria [8,13,14,15]. To date, approximately 2500 AMPs (natural and synthetic) are listed in the Antimicrobial Peptide Database (http://aps.unmc.edu/AP/main.php).

AMP microbicidal activity often relies on the electrostatic interaction between the positively charged peptide and the negatively charged microbial surface, which leads to AMPs accumulation and subsequent infiltration in the membrane [16]. According to this model, AMPs often present rapid killing kinetics together with a low tendency for selecting resistance [16]. The cationic nature of AMPs is also involved in their preferential binding to microbial membranes. In fact, many bacterial pathogen bilayers are highly electronegative, due to the presence of negative molecules (e.g., phosphatidylglycerol, phosphatidylserine), while mammalian membranes are usually enriched in zwitterionic phospholipids (e.g., phosphatidylcoline, phosphatidylethanolamine). Several negatively charged phospholipids, such as phosphatidylinositol and diphosphatidylglycerol, confer also to fungal membranes a negative net charge that attracts peptides. A further contribution to AMPs selective toxicity may be due to the different transmembrane potential (ΔΨ) between bacterial (from −90 to −110 mV) and host (−130 to −150 mV) cells. All these factors make mammalian membranes less prone to interact with AMPs [17].

AMPs may form permanent or transient pores in the lipid bilayer, as well as induce micelle release in a detergent-like manner, and/or cross the membrane without significant damage. [18,19,20,21]. Growing evidence indicates that AMPs mode of action may be actually influenced by additional factors, including pH, peptide concentration and salt abundance. Consequently, it is not unusual that a peptide that is reported to induce membrane permeabilization at high concentrations may reach intracellular targets without disrupting the lipid bilayer at lower concentration, or in different pH conditions [22,23,24].

The AMPs that do not directly target the cell membrane accumulate in the intracellular compartment, interfering with several essential processes and leading to: (i) impairment of DNA, RNA, protein or cell wall synthesis; (ii) interaction with intracellular enzymatic targets; (iii) release of oxygen species with consequent triggering of apoptosis and (iv) activation of signaling cascades following receptor-mediated uptake [22,23,24,25,26,27,28].

In addition to their antimicrobial activity, AMPs are known to be involved in immunomodulation. In fact, many AMPs have been proven to stimulate and/or hamper inflammatory response, induce cell migration and/or proliferation, and promote the wound healing process [7]. For this reason, AMPs are often referred to as *host defense peptides* (HDPs). In mammals, the mature protein is usually generated by proteolytic cleavage of a pro-peptide [10,12,29]. These genes are expressed in many cell types (e.g., monocytes/macrophages, neutrophils, epithelial cells and keratinocytes) either constitutively or following inflammatory stimuli [30,31]. In addition, the expression of many AMPs increases following injury and/or skin infection, accordingly to the role that this class of molecules plays in wound healing [32].

AMPs also play a plastic role in the modulation of the inflammatory response: they can recruit innate and adaptive immune effector cells (e.g., monocytes/macrophages, immature dendritic cells, neutrophils *etc.*), either directly or indirectly (by inducing chemokines/cytokines production), depending on their concentration [7,10,12,33]. On the other hand, AMPs can trigger the expression of anti-inflammatory genes and simultaneously shut down several pro-inflammatory pathways [12,34]. It has been hypothesized that this dual action may be involved in balancing the inflammatory process potentially triggered by commensal flora antigens [7].

Therefore, the broad-spectrum antimicrobial activity of AMPs, together with the plasticity of action at the interface between innate and adaptive immunity, make these molecules promising candidates for future therapeutic applications.

## 3. Hepcidins: A Conserved Class of Natural Peptides with Antimicrobial Activity

**Figure 1:**
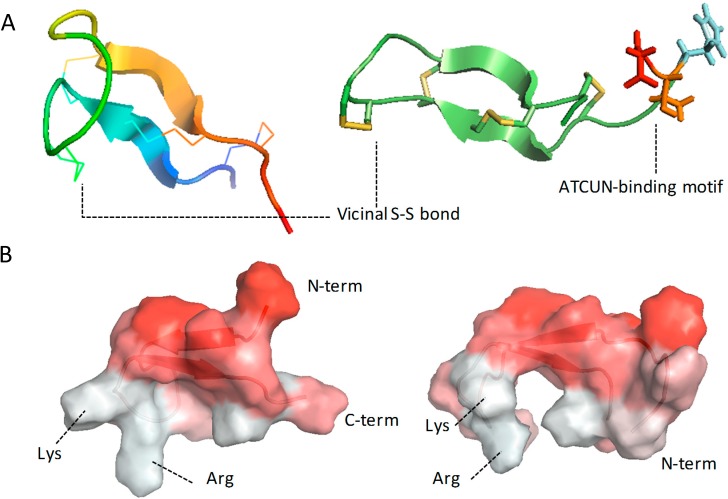
Molecular structure of hepcidin-20 and hepcidin-25. The cartoon representation of hep-20 structure is coloured in rainbow from the N- to the C-terminal portion, with disulfide bonds shown as lines (panel **A**, left side). Two distorted beta sheets are stabilised by 8 disulfide bonds, one of which notably occurs between adjacent cysteines at the hairpin turn (vicinal S-S bond). Hep-25 shows the same overall structure than hep-20, but it has 5 additional N-terminal residues; three of them (aspartic acid, threonine and histidine, coloured in red, orange, and cyan, respectively) are involved in the formation of the Cu^2+^-Ni^2+^ (ATCUN)- binding motif (panel **A**, right side). Panel **B** shows hep-20 and hep-25 (left and right side, respectively) surfaces coloured according to hydrophobicity (decreasing from red to white). Due to the distribution of the side chains, the convex face of both hepcidins is hydrophobic, whereas the concave one hosts positively charged residues, leading to a marked amphipaticity. Notably, the lacking of the first N-term residues reduces hep-20 hydrophobic character comparing to hep-25, making this form less prone to aggregation and therefore more active against microbes [37]. Molecular graphics created with PyMOL (The PyMOL Molecular Graphics System, Version 1.2r3pre, Schrödinger, LLC) using hep-20 and hep-25 NMR structures from the Protein Data Bank (www.rcsb.org; PDB file codes 1M4E and 1M4F, respectively, [37]).

The liver expressed antimicrobial peptide (LEAP-1) hepcidin is a natural host defence peptide produced by humans and originally detected in plasma and urine [35,36]. Two predominant forms have been isolated from urine, each composed of 20 and 25 amino acids and only differing by N-terminal truncation [35,36]. The structures of hepcidin-20 (hep-20) and -25 (hep-25) present a distorted β-sheet shape with a hairpin loop and are stabilized by disulfide pairing of Cys residues and hydrogen bonds between the two antiparallel strands (Figure 1).

This results in a marked amphipathic peptide structure, a conserved hallmark of many antimicrobial and antifungal peptides (Figure 1). The 84-amino acid hepcidin pre-pro-peptide contains a typical endoplasmic reticulum targeting signal sequence and a consensus cleavage site for pro-hormone convertases. The pre-pro-peptide is successively cleaved to yield pro-hepcidin (35aa) and the mature 25 amino acid hepcidin. Both the bioactive 25-aa hepcidin and the 35-aa pro-region are secreted by liver hepatocytes [38]. A metal-binding site is also present in the amino terminal region of the iron regulator hepcidin-25, the so-called Amino‒Terminus‒Copper‒Nickel binding site (ATCUN) motif, corresponding to the three first amino acids, which have been associated with the peptide ability to interact with ferroportin [39,40].

Human hepcidin has been demonstrated to act as an iron regulatory hormone, as well as to exert a wide spectrum antimicrobial activity [38]. Several homologues have been identified in other mammals, including horse, buffalo, camel, sheep, mouse [41,42,43,44,45], as well as in other vertebrates, such as fish, amphibians and reptiles [46].

A conserved sequence similarity over independent evolution of fish and mammals clearly indicates that this gene is vitally important for these organisms. It is known that mutations in the human hep-25 gene are related with pathologies such as human hereditary hemochromatosis [47], indicating that this peptide plays a prominent role in the regulation of iron concentration. In mammals iron sequestration is an innate host defence mechanism that limits the availability of this important element and prevents its uptake by invading microorganisms [46,47]. For example, it has been shown that horses experimentally infected with *Streptococcus zooepidermidis* show a decrease in serum iron levels, which drop before the onset of other clinical signs. Based on experimental data, equine serum iron levels have been used for monitoring the severity of inflammation [48,49].

Studies based on structural similarity between hepcidin from mammals and other organisms along the evolutionary scale, have led to the hypothesis that the iron-regulatory hormone has most likely evolved from an antimicrobial peptide during vertebrate evolution [50]. Indeed, the antibacterial activity was the first clue leading to the discovery of hepcidin peptides, which highlighted the wide spectrum activity *versus* many pathogenic bacteria and disclosed their important role in the innate immune response of fish [50,51].

**Figure 2:**
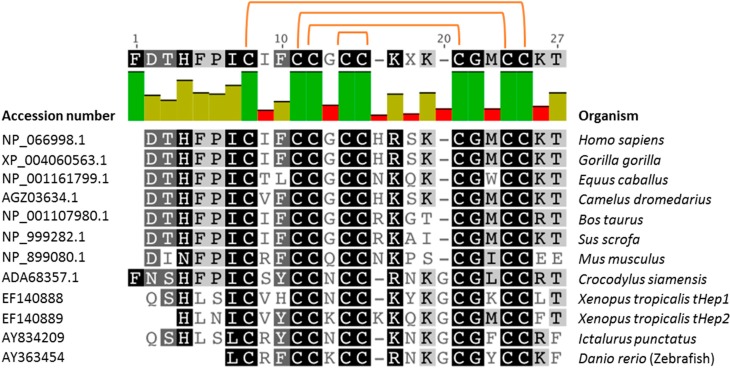
Comparison of mature hepcidin amino acidic sequence in different species. Sequence alignment was based on GeneBankTM accession numbers using Geneious software (http://www.geneious.com) [53]. Percent identity is color coded: bright green represents 100% identity, green-brown represents 30%–100%, while red indicates an identity score below 30%). Position of the four highly conserved disulfide bridges is indicated with orange lines, connecting the eight involved cysteine residues.

A conserved role in the immune response of organisms along the evolutionary scale implies structure similarities conserved and shared by different hepcidin homologues isolated from different organisms. As shown in Figure 2, the primary structures of mature hepcidin from higher and lower vertebrates share six to eight conserved cysteine residues in key positions, indicating that the disulfide bridges of hepcidin have been evolutionarily conserved and may relate to peptide biological function [52].

In this respect, it has been shown that intact disulfide bonding pattern is not essential for the regulation of iron homeostasis by human hep-25, but rather it plays an important role in the peptide stability in biological fluids [52].

## 4. Antimicrobial Activity of Human Hepcidins

### 4.1. Antibacterial Activity

The antibacterial properties of human hepcidins were described for the first time by two groups, using different antimicrobial susceptibility techniques [35,36]. Using a radial diffusion assay Krause and coworkers observed a dose-dependent inhibitory effect of hep-25 against the Gram-positive species *Bacillus megaterium*, *B. subtilis*, *Micrococcus luteus*, *Staphylococcus carnosus*, and the Gram-negative species *Neisseria cinerea* [35]. A colony count assay in sodium phosphate buffer was used by Park and coworkers to evaluate the bactericidal activity of hep-25 and its analogue hep-20 [36]. Both peptides exhibited antimicrobial properties against *Escherichia coli* and, to a lesser extent, against *S. epidermidis*, *S. aureus*, and group B streptococci [36]. More recently, we investigated the antibacterial activity of both hep-25 and its analogue hep-20 against a wide array of Gram-positive and Gram-negative multi-drug resistant clinical isolates [23]. Both hep-25 and hep-20 were found to be bactericidal at concentrations ranging between 3.25 and 50 μg/mL, depending on the bacterial species tested [23]. Among all the bacterial species tested, *S. aureus* was the one exhibiting the highest degree of resistance to both hepcidins, suggesting an inherent insensitivity of this microorganism to such peptides. Interestingly, the antimicrobial potency of hep-20 was found to be higher than that of hep-25 [23], being the peptide bactericidal at lower concentrations than hep-25 against most of the bacterial species tested. Killing kinetics of both peptides carried out at neutral pH against *Pseudomonas aeruginosa* and *Enterococcus faecium* indicated that hep-20 exerted a bactericidal effect in a time interval ranging between 30–60 min, while hep-25 resulted bactericidal not before 60–90 min. Notably, non-bactericidal concentrations of hep-20 and hep-25 combined together showed a synergistic antibacterial effect against *E. faecium* [23], suggesting that the two peptides may cooperate *in vivo* in the innate defense mechanisms to infections. In this regard, it has been proposed that the generation *in vivo* of hep-20 may not simply reflect the catabolism of hepcidin, but rather led to the formation of a peptide endowed with more potent antibacterial activity compared with hep-25 [23].

Antimicrobial properties of the 35 aa hepcidin pro-region have also been demonstrated [38]. In particular, this peptide was found to be bactericidal against *B. megaterium* (25 µM), and showed a bacteriostatic effect against *B. subtilis*, but only at high concentration (200 µM) [38].

### 4.2. Role of the ATCUN Motif

The N-terminal region of hep-25 represents a metal binding site specific for Cu(II) and Ni(II), known as “ATCUN” motif [54]. Although the biological role of a metal binding motif in hep-25 is still unknown, its presence is physiologically relevant for the hep-25 iron regulation properties as the first five amino acids, starting from the N-terminus, are essential for its interaction with ferroportin [55]. It is known that the antibacterial activity of some human peptides (e.g., histatin-5, peptidoglycan-recognition-protein) is enhanced by the presence of metals, although the underlying mechanisms are still unknown [56,57]. To assess the hypothesis that the metal-binding site on hep-25 could contribute to the bactericidal activity of the peptide we performed antimicrobial susceptibility tests in the presence of CuCl_2_. By sequentially exposing *S. aureus* and *P. aeruginosa* cells to hep-25 and then to physiological concentration of CuCl_2_, but not co-incubating the bacteria with hep-25 and CuCl_2_, a statistically significant enhancement of the bactericidal activity of the peptide against both species was observed [23]. When the same experiments were carried out using hep-20 no increase of the bactericidal activity was observed, supporting the hypothesis that the interaction ATCUN motif—metal does play a role in enhancing the antimicrobial activity of hep-25 [23]. The possible mechanism by which CuCl_2_ may exert such an effect is not currently known. Interestingly, an ATCUN motif is also present in other human histidine-rich peptides (e.g., histatin-5), and it has been reported that high levels of hydrogen peroxide are produced in aqueous solutions containing Cu(II), histatin-5, and a reductant, either ascorbate or cysteine [54,58,59]. This suggests that the presence of an ATCUN motif may confer to the corresponding peptides the ability to generate reactive oxygen species in the presence of metals. Assuming that hep-25 is able to cross the bacterial membranes (see below) and reach the intracellular milieu, it might be hypothesized that the peptide forms complexes with copper and, in the presence of intracellular reductants, may give rise to reactive oxygen intermediates. These, in turn, may induce DNA hydrolysis as well as cross-linking and cleavage of bacterial proteins, possibly enhancing the antibacterial activity of hep-25 [54,58,59,60].

### 4.3. Role of Disulfide Bonds

In human hepcidins disulfide bonds have a fundamental role in maintaining the correct folding of peptides [60]. Stabilization of the three-dimensional structure by the intra-molecular disulfide bridges seems to be also crucial for the antibacterial activity of these peptides [52]. In fact, when all the cysteine residues of hep-25 were replaced by alanines the peptide lost its antibacterial activity [52]. This observation is in contrast with what has been reported for other peptides for which the disulfide bonds do not play a role or even represent an obstacle for their antibacterial performances [61,62]. For instance, the antimicrobial and cytotoxic activities of the human beta-defensin 3, a peptide with structural similarities with hepcidins, are independent on whether and how cysteine residues are arranged to form disulfide bonds [61]. In the case of the human beta-defensin 1, only the reduction of the disulfide bridges allows the peptide to exert potent antimicrobial properties. In fact, reduced hBD-1 differs structurally from oxidized hBD-1 and the free cysteines at the carboxy terminus seem important for the bactericidal effect of the peptide [62].

### 4.4. DNA Binding Ability

It has been demonstrated that hep-25 is able to bind efficiently DNA, even in the presence of high concentrations of salts [39,52]. Hep-25 DNA-binding ability seems dependent on the integrity of its disulfide bridges as their reduction or replacement of the eight cysteines of the peptides by alanine residues led to peptides that are no longer able to bind DNA in *in vitro* assays [52]. A similar observation was also obtained with the hepcidin pro-region that showed a DNA binding ability similar to that observed for magainin 2, an AMP known to exert its antimicrobial effect by interfering with intracellular nucleic acids [52].

### 4.5. Enhancement of Bactericidal Activity in Acidic Conditions

One of the most interesting aspects that has emerged from studies examining the antibacterial properties of both hep-25 and hep-20 is the net increment of their bactericidal effect in acidic conditions [23]. In particular, from 2 to 8 fold reduction of bactericidal concentrations of both hepcidins was observed at acidic pH (6.6, 5.8, and 5.0) compared to pH 7.4, paralleling the increment of theoretical net charge of the peptides along with pH reduction. Acidic pH (pH 5.0) not only reduced the microbicidal concentrations of both hepcidins against both Gram positive (*E. faecium*) and Gram-negative (*P. aeruginosa*) bacterial species, but also markedly shortened the killing times of both peptides compared to pH 7.4 [23]. It is worth noting that by combining very low concentrations of hep-20 and hep-25 at pH 5.0 a bactericidal effect could be obtained at peptide levels very close to those measured in human serum (range 0.1–4 μg/mL) and urine during infective/inflammatory processes [63,64], supporting the hypothesis that hep-25 and/or hep-20 may have *in vivo* antibacterial functions, other than iron regulation properties [23].

It has been reported that the presence of histidine residues in the amino acidic sequence of a peptide increases its antimicrobial activity at acidic pH [65]. Thus, the marked enhancement of the bactericidal activity of hep-20 and hep-25 at pH 5.0 could be due to the presence of histidine residues in the amino acid sequence of these peptides. At pH values below pKa of the histidine groups (pKa = 6.5) these residues may be protonated, resulting in an increase of the net charge of the corresponding peptide. As also suggested for other peptides [65,66,67,68], this protonation could facilitate the electrostatic interaction of hepcidins with the anionic microbial surfaces and, consequently, improve their antibacterial properties. It has been reported that the solubility of a linear form (reduced) of hep-25 is higher at acidic pH (5.5) than at neutral pH, suggesting that at low pH values hepcidins may exhibit poor tendency to aggregate with consequent increment in the number of correctly folded molecules, which, in turn, may result in an enhanced antimicrobial activity [60].

Recently, we investigated the influence of pH on the mode of action of hep-20 and hep-25 against *E. coli* ATCC 25922 and model membranes [24]. By using several experimental approaches, we demonstrated that while at acidic pH the peptides cause a rapid and marked permeabilization of bacterial and model membranes, no inner membrane perturbation capacity was observed at pH 7.4, even at bactericidal concentrations. Scanning electron microscopy studies revealed a different pattern of morphological effects caused by both peptides on *E. coli* cells at neutral and acidic pH. In particular, a much higher ability of the peptides to induce bleb formation on the bacterial surface at pH 5.0 than at pH 7.4 was observed. This ability has been previously reported for other AMPs such as SMAP-29 [69], magainin 2 [70] or temporin L [71] able to act by perturbing the membrane integrity. Based on the data obtained in these studies, we hypothesized two not necessarily mutually exclusive hepcidin mechanisms of action: at neutral pH, the low membrane-perturbing capacity seen at bactericidal concentrations may suggest that an intake of the peptides followed by their interaction with one or more intracellular molecular targets (e.g., DNA) is the predominant hepcidins’ mode of action. On the other hand, the striking membrane damaging ability exhibited by both peptides at acidic pH, suggests that a classical membrane perturbing mechanism is mainly responsible for cell death at pH 5.0. This hypothesis is supported by the observation that at pH 5.0 both hepcidins were able to reduce the number of viable *E. coli* cells by 3 log in less than 5 min. Such a rapid killing effect is typical of peptides with a membrane targeting mode of action rather than of peptides acting on intracellular targets that exhibit, in general, relatively slow bacteria-killing kinetics (from several minutes to some hours) [72]. These findings suggest that the mechanism of action of human hepcidins may vary according to pH and may help in the development of hepcidin-derived peptides for the local treatment of infections occurring in body districts with acidic pH.

### 4.6. Antifungal Activity

The resistance to antifungal therapy observed in some species, either intrinsic or acquired in course of treatment, currently represents a major concern in the management of mycoses, thus boosting the research for new potential antimicrobial compounds [73]. Hepcidins were first described to possess an antifungal activity by Park and co-workers, who demonstrated a direct killing activity against *Candida albicans* and molds like *Aspergillus fumigatus* and *A. niger* [36].

The last two decades saw a rise in *Candida glabrata* prevalence, possibly due to the widespread use of fluconazole, to which this species is less sensitive than *C. albicans* and other non-albicans species [74]. This emerging pathogen is responsible for both systemic and mucosal infections, being now second in frequency only to *C. albicans* [75,76]. Although many AMPs (e.g., histatins, cathelicidins, defensins, and magainin) have been proven to exert antifungal activity *versus* clinically relevant fungal pathogens, *C. glabrata* seems to be scarcely susceptible to these molecules [77,78,79,80,81].

Interestingly, we demonstrated that hep-20 exerts *in vitro* fungicidal effect on a panel of *C. glabrata* clinical isolates with different fluconazole susceptibility [82], while hep-25 did not show any killing activity against this species (unpublished results). Following a 90-minute incubation in sodium phosphate buffer at pH 7.4, hep-20 minimal fungicidal concentration (MFC) ranged from 50 to 100 μM depending on the strain [82]. Despite the fact that these concentrations are higher than bactericidal ones, they are actually comparable to those reported for other antifungal peptides [83]. Most importantly, hep-20 effect on *C. glabrata* seems to be enhanced in acidic condition, as already reported for its antibacterial activity [23]; indeed, the MFC drops to 25 μM for most strains tested at pH 5.0. Moreover, under acidic condition the killing time of the peptide was also shortened, with a fungicidal effect occurring between 30 and 60 min, compared to kinetics at pH 7.4, where it occurred not earlier than 60–90 min [82]. Since a further interesting characteristic of many AMPs is their ability to act synergistically with traditional antimicrobial compounds, the effect of hep-20 in combination with amphotericin B, caspofungin or fluconazole in acidic condition was also evaluated. Non-fungicidal combination of hep-20 and amphotericin B resulted in a significantly enhanced fungicidal effect in comparison with the most active component alone, after 24-hour incubation [82]. Similarly, an increase in fungicidal activity could be observed for hep-20/caspofungin combination, with a synergistic effect occurring at 90 min and being maintained later on [82]. A synergistic effect of the hep-20/fluconazole combination could be detected too (in sub-inhibitory concentration each, 50 μg/mL hep-20 + 64 μg/mL fluconazole), as stated by a FICI value of 0.5 [82].

The enhanced activity of hep-20 observed under acidic conditions suggested that this peptide could be used as a therapeutic agent in the topical treatment of recurrent vulvovaginal candidosis due to *C. glabrata* [75,84]. In addition to the scarce susceptibility to azole drugs commonly used in the treatment of mucosal infections, a rising number of both fluconazole and echinocandins resistant isolates of *C. glabrata* has been recently documented [85,86]. As a result, vaginal infections due to this species are difficult to eradicate and often end up in recurrent episodes, thus highlighting the need for new therapeutic strategies [75]. As already mentioned, despite all the interesting features of AMPs, there are several limitations holding back their application, including the significant reduction of their activity in biological fluids due to physiological concentrations of salt [87,88,89,90].

Accordingly, it was not surprising that hep-20 activity was found to be impaired in a vaginal fluid simulant (VFS) resembling human secretion; in this condition, only the highest peptide concentration tested induced a significant reduction in *C. glabrata* CFU number, even if it did not reach a fungicidal effect. However, the addition of the chelating agent EDTA (1 mM) to VFS significantly enhanced hep-20 activity, thus confirming a role for cations in hep-20 activity depletion. To strengthen the hypothesis of a potential application of hep-20 in the topical treatment of vaginitis due to *C. glabrata*, the fungicidal activity of this peptide has been evaluated in human vaginal fluid (HVF) collected from three healthy donors [91].

The antifungal activity of hep-20 in HVF seems to be influenced by the biological variability occurring between the donors. In fact, while in the case of one donor no antifungal activity was observed following a 90-minute incubation at 37 °C, a significant reduction in the number of viable yeasts was induced by hep-20 for the other two donors. This could be possibly due to the presence of endogenous AMPs (e.g., calcoprotectin, HBD1-2, HNP1-3, and LL-37) synergizing with hep-20, thus overcoming the inhibitory effect caused by ions or other fluid components [91,92,93,94]. Notably, following the addition of 1.5 mM EDTA to the HVF, hep-20 exerted a complete fungicidal effect on *C. glabrata* at a concentration that was a quarter of the one that significantly reduced cell number in VFS for all donors [91]. Overall, these encouraging results obtained in HVF support a role of hep-20 as a promising candidate, which could be further investigated for the topical treatment of *C. glabrata* vaginal infections.

## 5. Hep-20 Cytotoxicity and Stability

Even though hep-20 is a human-derived peptide, therapeutically active concentrations could be higher than those found at physiological levels. Therefore, it was essential to investigate the potential cytotoxic effects exerted by this peptide on human cells. Our data report a lack of hemolytic effect on human erythrocytes [82]. Moreover, hep-20 cytotoxicity was evaluated by measuring the effect of different peptide concentrations on human PBMC and a human-derived epithelial cell line (A549) by PI staining and XTT reduction assay. All hep-20 concentrations tested (6.25 to 100 μM) gave negligible or no cytotoxic effect on peripheral blood mononuclear cells and A549 cell line, as demonstrated by PI staining and XTT assay [91]. These results are in agreement with a study by Park and coworkers in which hep-20 was tested for cytotoxicity on erythroleukemia type K562 human cells (88% viable cells post treatment) [36]. For what concerns the stability of the peptide in human fluids, no information is available to date on hep-20 half-life in humans. However, indirect evidence on hep-25 clearance from human plasma has been obtained, revealing that hep-25 levels decrease over time, with a mean plasma elimination half-life ranging between 5 and 8 h [95].

## 6. Inhibition of Biofilm Formation

Biofilms are defined as complex communities of microorganisms living attached to biotic or abiotic substrates and enclosed in a self-produced polymeric extracellular matrix [96]. It is now clear that biofilm formation is responsible for a large proportion of infections in developed countries, especially in the hospital setting [97]. Examples of biofilm-associated infections are periodontitis, medical device-related infections, pneumonia in cystic fibrosis patients, chronic urinary tract infections, recurrent tonsillitis, chronic rhinosinusitis, otitis media or wound infections [98]. Biofilm-associated infections represent a serious health-care concern, as they are difficult to prevent, diagnose, and treat [97]. In particular, biofilm treatment is particularly challenging as biofilms usually exhibit a severely reduced susceptibility to antimicrobials due to several mechanisms. These include: reduced diffusion or sequestration of antimicrobials through the extracellular matrix, low growth rate of biofilm cells, presence of dormant cells virtually tolerant to all drugs [96].

Due to the peculiar features of biofilm communities, the identification of new anti-biofilm drugs, able to specifically target the biofilm mode of growth, is highly needed. In this regard, the possible use of AMPs as new anti-biofilm agents is an emerging area of research [99,100]. A large number of AMPs with different structures and physical-chemical properties have been tested against bacterial/fungal biofilms with promising results [101,102,103,104].

An updated list of biofilm-active AMPs and related references can be found at www.baamps.it website, which was recently developed with the objective to collect data present in the literature on AMPs specifically tested against microbial biofilms [105].

One of the leading nosocomial bacterial pathogens, whose pathogenicity is significantly linked to its ability to form biofilms on medical devices (e.g., central venous catheters, artificial heart valves, or prosthetic joints), is *Staphylococcus epidermidis*. *S. epidermidis* biofilms form according to a well-defined multistep process that involves attachment to a surface, production of accumulation factors that allow cell-to cell adhesion, maturation, and detachment of single or clustered cells with colonization of surrounding sites or invasion of blood stream [106]. One of the best studied *S. epidermidis* accumulation factors is the polysaccharide-intercellular-adhesin (PIA), a polymer of β-1,6-linked N-acetyl-glucosamine with partially N-deacetylated amine groups, whose synthesis and secretion is under the control of the *ica* operon [106]. Recent epidemiological studies have reported that even PIA-negative strains might exhibit marked biofilm forming ability, with proteins being the main accumulation factor in these strains [107].

The antibacterial properties of hep-20 have recently been investigated *in vitro* against biofilms of a number of *S. epidermidis* strains isolated from central venous catheters or blood [108]. All strains were finely characterized for their ability to produce biofilms with extracellular matrix mainly constituted of PIA or proteins. A striking ability of hep-20 to inhibit biofilm formation of all the strains, in a dose dependent manner, was observed, with no major difference between PIA-positive and PIA-negative strains. Interestingly, the anti-biofilm activity of hep-20 was strengthened at the mild acidic pH found on the human skin (pH 5.5) as compared to pH 7.4, although pH enhancement of peptide’s anti-biofilm activity was far less evident than that previously observed against floating bacteria [23]. Kinetics studies showed that the inhibition of biofilm formation by hep-20 started already after 3 h of incubation with *S. epidermidis* cells and that the inhibitory effect continued for at least 16 h after peptide removal [108]. The reduction in biofilm biomass of *S. epidermidis* biofilms exerted by hep-20 paralleled a reduction in biofilm metabolic activity, while had only a marginal effect on biofilm-associated viable cells.

Little is known about the mechanisms of the anti-biofilm action of AMPs, but what is progressively emerging is that many AMPs may act with mechanisms that differ by a “classical” microbicidal effect [99,109]. These include: (i) inhibition of the initial phase of bacterial adhesion to a surface; (ii) stimulation of motility; (iii) inhibition of quorum sensing, the bacterial communication machinery that regulates many virulence traits, including biofilm formation; (iv) modulation of gene expression. Of note, the anti-biofilm activity of hep-20 was obtained at concentrations far below the MIC (minimal inhibiting concentration) values of the peptide suggesting that it could act by a “non classical” mode of action. Confocal microscopy studies demonstrated that, in the presence of hep-20, both PIA-positive and PIA-negative *S. epidermidis* strains developed biofilms with an altered architecture and reduced amount of extracellular matrix [108]. Interestingly, pre-incubation of *S. epidermidis* biofilms with hep-20, followed by incubation with sub-inhibitory concentrations of vancomycin, caused a statistically significant reduction of biofilm-associated viable cells of both PIA-positive and PIA-negative strains, as compared to biofilms not pre-treated with hep-20. Overall, these results suggest that hep-20 may exert its anti-biofilm activity by interfering with extracellular matrix accumulation and this, in turn, may improve diffusion of conventional antibiotics (e.g., vancomycin) through the biofilm layers, pointing to a possible use of the peptide in anti-biofilm combinatorial therapies.

## 7. From the Bench to the Clinical Setting: Advantages and Obstacles

The direct application of AMPs to the clinical setting has been hampered due to poor bioavailability, pharmacokinetics and high synthesis/manufacturing costs [110]. In order to overcome AMP weaknesses several strategies have been developed, including minimizing the peptide length or converting the original peptide into a peptidomimetic, a small protein-like chain with an altered chemical structure appropriately designed to increase stability or biological activity of the peptide and to reduce the peptide susceptibility to proteases [111,112]. Information on the selected peptide biochemical, biological and pharmacological properties is an essential prerequisite for modifying the native peptide into a promising novel drug. To this end, computer-assisted AMP design programs provide an accurate prediction of the “cidal” effect possessed by the modified peptide or peptidomimetic [113]. Nevertheless, in the case of hepcidins, the activity of which is strongly dependent upon disulfide bridges, this might not be the ideal choice, since it could be difficult to predict the exact rate of correct folding of the synthetic peptide. In addition, disulfide rich peptides, like hepcidins, are difficult to prepare, since they require extensive redox folding procedures to achieve the creation of four disulfide bridges. Moreover, they tend to aggregate, giving rise to a poor yield.

Other strategies include the multimerization of AMPs, where multiple copies of a given peptide are assembled around a core molecule, with the aim of increasing the peptide activity [114]. Multivalent peptides have been shown to possess strong antifungal properties [115,116].

Encapsulation of AMPs in small drug carriers offers the double effect of protecting the peptide from premature degradation, and of ensuring a local delivery of the compound. In recent years, nanocarriers, such as liposomes, dendritic polymers, solid core nanoparticles, carbon nanotubes have been developed, which are able to be captured by several cell types [117] and can be engineered to deliver the appropriate peptide content with a targeting moiety carefully designed for different applications [118,119,120]. This could represent a promising approach for a tailored delivery of hepcidin into the host.

Finally, another interesting approach under evaluation is the possibility of using AMPs as coating agents to prevent colonization/infection of medical devices. It is renowned that catheter-associated infections are often caused by microbial colonization on prosthetic materials, which in turn hampers an efficient eradication of the infection. In this respect, biofilm producing microorganism create an ideal niche for their survival, with the extracellular matrix protecting sessile cells and often contributing to the development of a drug resistant phenotype. Hep-20 could represent promising agent in inhibiting biofilm formation on plastic surfaces, since it is small enough to penetrate the exopolysaccharide matrix, has a wide spectrum activity and good biocompatibility. The previously reported ability of hep-20 to interfere with extracellular matrix accumulation of relevant biofilm forming microorganisms looks promising for the identification of future combinatorial therapies of the peptide with bactericidal compounds aimed at eradicating biofilm on prosthetic materials.

## 8. Hepcidin 20 as a Potential Drug for Future Use

The rise in drug resistance among clinically relevant microorganism has triggered the study of alternative therapeutic strategies. AMPs are considered promising candidates for the development of novel antibiotics. Among these, human hepcidins show an interesting potential as antimicrobial agents, active on both bacterial as well as fungal species. The enhancing effect of low pH on bactericidal activity of human hepcidins render these peptides interesting for the development of future drugs specifically designed for the treatment of bacterial and/or fungal infections occurring in body districts with acidic pH. From this point of view, hep-20 looks particularly promising as, unlike hep-25, the peptide does not seem to be involved in iron homeostasis and exerts antimicrobial activity at concentrations generally lower than hep-25 [23]. In addition, hep-20 has shown activity against multidrug-resistant fungal strains [82] that were insensitive to the inhibitory effects of hep-25 (unpublished results). Both physiological and pathological mechanisms are responsible for generating a low pH *in vivo*. For instance, healthy skin has a normal pH of about 5; it is well established that acidic pH plays a role in skin defense against fungal and bacterial infections [121] and that skin disorders, associated with increased cutaneous pH, predispose patients to secondary microbial infections. The vagina of reproductive age women is another district physiologically maintained at acidic pH (4.0–5.5) by the presence of *Lactobacillus* spp. and other bacteria that produce lactic acid by sugar fermentation [122]. Although differences in species composition and relative-abundances of populations in vaginal bacterial communities have been described across women from various ethnic backgrounds, low pH is a highly conserved feature and it is believed to contribute in restricting the growth of pathogens and other opportunistic organisms [122].

The generation of a low pH consequent to the fermentation of dietary sugar by oral bacteria is considered a key step in the pathogenesis of dental caries by selecting aciduric bacterial species involved in cariogenesis [123]. The urinary tract of most individuals, the gastric mucosa, or the mature phagosome following phagocytosis of microbes are all further examples of environments characterized by acidic pHs.

Interestingly, it has been recently demonstrated that hepcidin is localized in gastric parietal cells, regulates acid secretion and is induced by *Helicobacter pylori* infection [124]. Of note, knockout mice with hepcidin-deficiency demonstrated an impaired acid secretion by gastric cells and experienced gastric bacterial overgrowth [124]. This finding leads to the intriguing hypothesis that individuals genetically less prone to hepcidin production may be more susceptible to *H. pylori* gastric infection and that therapeutical administration of hepcidin in such subjects may restore acid secretion and inhibit bacterial growth.

Another important observation was made by Sow and coworkers, who reported a synergistic induction of hepcidin in bone marrow derived mouse macrophages treated with IFN-γ and *Mycobacterium tuberculosis* [125]. The same authors demonstrated that hepcidin localizes to the mycobacteria-containing phagosomes and that the peptide possesses an antimicrobial activity against *M. tuberculosis in vitro* [125]. As previously mentioned, the design of hep-20 peptides, chimeric for motives driving intracellular penetration (cell-penetrating-peptides) [126], could be an interesting field of investigation aimed at the development of new generation of drugs targeting intra-phagosomal bacteria.

**Figure 3:**
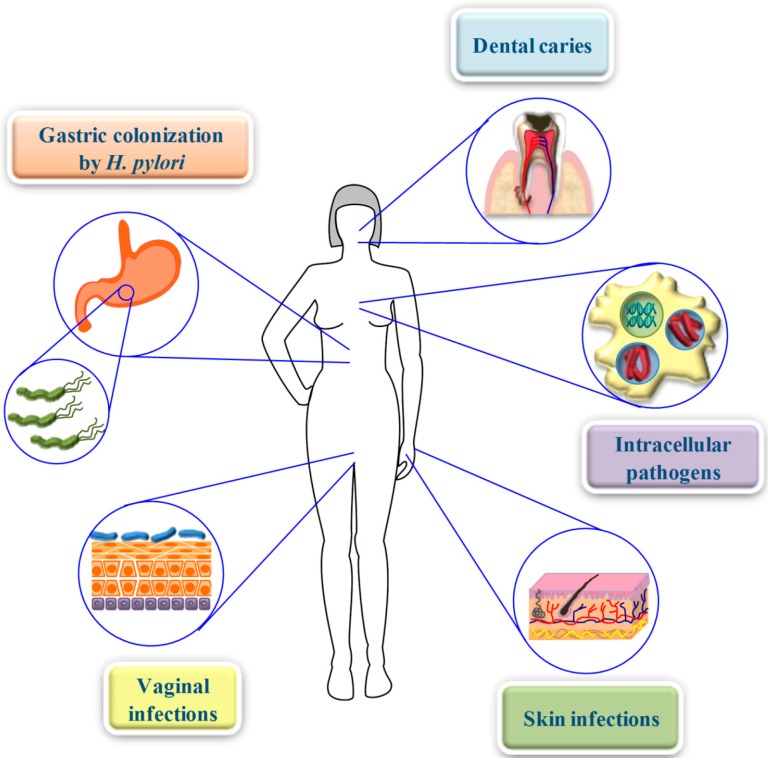
Body districts characterized by an acidic pH, where hep-20 could express its potentiated antimicrobial properties in the control of both bacterial and fungal infections.

Thus, design of hep-20 based peptides, could be a promising strategy to treat a number of infections in disparate body districts and conditions (Figure 3) in view of:

(i) the high cytocompatibility of such peptides [82,91],
(ii) the ability to specifically be more active at acidic pH [23,24,82,91],
(iii) the possibility to act synergystically with other endogenously produced peptides [91],
(iv) their activity against a wide range of multidrug-resistant bacterial and fungal strains [23,82,91].

Nevertheless, difficulties in synthesis, due to the need of disulfide bridges for maintenance of antimicrobial activity [51], partial inhibition by body fluids [90], and relatively high active concentrations, represent possible obstacles for the use of hepcidin-derived peptides as future drugs, demanding further bench research for their optimization and use in the clinic as new therapeutics.

In conclusion, although their clinical application is still hindered by technical obstacles, hepcidin modification or innovative delivery formulation will hopefully help us crossing the bridge still existing from the bench to the clinic bedside.
